# The Role of Selenium as an Antioxidant in the Treatment of Renal Cell Carcinoma

**DOI:** 10.7759/cureus.98696

**Published:** 2025-12-08

**Authors:** Ziad El Menawy, Hussein Hmoud, Mina Taher, Taqwa Atef, Ibrahim Elsharkawi, Anu V Ranade

**Affiliations:** 1 Trauma and Orthopedic, University Hospital of Wales, Cardiff, GBR; 2 Paediatric Burns and Plastic Surgery, Royal Manchester Children’s Hospital, Manchester, GBR; 3 Trauma and Orthopaedics, Manchester Royal Infirmary, Manchester, GBR; 4 Internal Medicine, Zayed Hospital, Abu Dhabi, ARE; 5 Endocrinology, William Harvey Hospital, East Kent, GBR; 6 General Practice, East Kent Hospitals NHS Foundation Trust, East Kent, GBR; 7 Pediatrics, Al Jalila Children's Hospital, Dubai, ARE; 8 Research and Development, University of Sharjah, Sharjah, ARE; 9 Basic Sciences, University of Sharjah, Sharjah, ARE

**Keywords:** cancer therapy, hydrogen peroxide (h2o2), reactive-oxygen-species, renal cell carcinoma (rcc), selenium

## Abstract

Background: Antioxidants are said to have a major role in preventing cancerous growth, and selenium, being one of the vital trace elements, plays a crucial role in the oxidation-reduction system. Selenium, however, demonstrates a well-recognised dose- and context-dependent dual behaviour: while acting as an antioxidant at physiological levels, it may exert pro-oxidant and cytotoxic effects at higher concentrations, a phenomenon previously described in cancer biology. In parallel, reactive oxygen species (ROS), intracellular oxidants such as hydrogen peroxide, are key mediators of oxidative stress that influence cancer cell survival and death. Given this interplay, the role of selenium in modulating oxidative stress remains controversial.

Aim: To explore the effects of selenium as an antioxidant on renal cell carcinoma (RCC).

Methods: RCC cell lines under strict culture conditions were divided into two groups: control (group A) and experimental groups. Experimental groups were selenium treated (group B), exposed to oxidative stress induced with H₂O₂ (group C), and exposed to oxidative stress induced with H₂O₂ followed by selenium treatment (group D) and selenium treatment followed by oxidative stress induced by H₂O₂ (group E). Cell growth and survival were evaluated in each group after 72 hours. Evaluation of the ROS was done using the Cellular Reactive Oxygen Species Detection Assay Kit (Abcam; ab186029), and cell viability was measured using MTT reagent (Sigma; 11465007001).

Results: The generation of the ROS was significantly high in groups C (125.4% ± 14.6), D (132.9% ± 16.6), and E (135.3% ± 22.1) when compared to groups A (100% ± 14) and B (91.7% ± 14.6). Moreover, the RCC cell viability was 98.7% ± 10.8 in group B compared to C, D and E (80.8% ± 5.4, 79.7% ± 5.9 and 74.8% ± 8.8, respectively). Moreover, an association was observed between the viability of cells and ROS generation. The higher the ROS generation (groups C, D, and E), the lower its viability. Likewise, the lower the ROS generation (group B), the more viable were the cells.

Conclusions: the effects of selenium as an antioxidant in promoting cell viability in renal cell carcinoma have been controversial. In some aspects, it reduced ROS production, which increased cancer cell viability. While in another setting, selenium increased ROS levels, which correlated with more cell death. Therefore, further research needs to be conducted to prove the true effects of selenium on cancer cells.

## Introduction

Reactive oxygen species (ROS) are natural byproducts of cellular oxygen metabolism. Under stressful conditions or disturbances in redox homeostasis, ROS can accumulate to harmful levels, damaging cellular organelles, impairing homeostasis, and contributing to carcinogenesis [1,2]. Cancer cells are known to maintain elevated baseline ROS levels due to accelerated metabolism, mitochondrial dysfunction, oncogenic signalling, and rapid proliferation. Although moderate ROS concentrations support tumour growth by activating pro-survival pathways, excessive ROS can overwhelm antioxidant defences, causing oxidative damage to DNA, proteins, and lipids, ultimately triggering cell-cycle arrest or cell death. Thus, the balance between ROS production and antioxidant capacity is a critical determinant of cancer-cell behaviour.

Within this framework, some theories propose that administering antioxidants may inadvertently stabilise cancer cell survival by lowering ROS below cytotoxic thresholds, potentially worsening prognosis in certain malignancies. Conversely, persistently elevated ROS levels in highly proliferative cancers can induce oxidative DNA damage and apoptosis, thereby suppressing tumour growth. Selenium is particularly relevant in this context because it can function as either an antioxidant or a pro-oxidant depending on dose, chemical form, and cellular environment. This highly dose- and context-dependent dual behaviour has been well described in cancer biology and contributes to the ongoing debate regarding selenium’s impact on tumour progression.

Previous studies have shown that antioxidants play a major role in preventing cancerous growth, and selenium, through its incorporation into various selenoproteins, has been considered an important contributor to antioxidant defence [3]. However, selenium has also been characterised as a “double-edged sword”, exhibiting antioxidant effects at physiological levels while exerting pro-oxidant, DNA-damaging, and cytotoxic effects at higher concentrations or in specific tumour microenvironments. Some reports further suggest that providing antioxidants to cancerous tissues could blunt ROS-mediated cytotoxicity, thereby protecting tumour cells and worsening outcomes [4-7]. Therefore, the precise role of selenium in modulating oxidative stress and influencing tumour behaviour remains controversial.

In renal cell carcinoma (RCC), the role of selenium remains insufficiently defined. Clinical studies have reported altered selenium levels and dysregulated selenoprotein expression in RCC patients, suggesting that selenium-dependent redox pathways may influence tumour development and progression. Experimental work in RCC models has demonstrated that selenium compounds can exert pro-apoptotic effects through ROS generation at higher doses, whereas lower concentrations may enhance antioxidant defences and support tumour cell viability. These mixed and sometimes opposing findings mirror the broader controversy surrounding selenium biology in oncology and highlight the lack of consensus regarding whether selenium ultimately promotes or suppresses RCC growth.

This study therefore investigates whether selenium exposure in RCC cells promotes cell death or enhances survival, thereby clarifying selenium’s direction of effect in this specific tumour type.

## Materials and methods

Caki-2 (ATCC; HTB-47) Clear cell carcinoma cell line, Dulbecco Modified Eagle’s medium DMEM (Gibco; 61965), 10% foetal bovine serum (Sigma; F7942), penicillin (100 U/ml) / streptomycin (100 µg/ml) antibiotic mixture (Gibco; 15140), 1x trypsin/EDTA (Gibco; 15400), flat-bottom 96-well plate, hydrogen peroxide solution (Sigma; H1009), selenium (Sigma-Aldreich; 229865), thiazolyl blue tetrazolium bromide (Sigma; M2128), Cellular Reactive Oxygen Species Detection Assay Kit (Abcam; ab186029), and assay buffers.

Cell culture

Caki-2 (ATCC; HTB-47), a clear cell carcinoma cell line frozen in liquid nitrogen, was obtained (Gibco; 61965). The cells were maintained in Dulbecco's Modified Eagle's medium DMEM (Gibco; 61965) supplemented with 10% foetal bovine serum (Sigma; F7942) and penicillin (100 U/ml) / streptomycin (100 µg/ml) antibiotic mixture (Gibco; 15140). Then, the cells were later dissociated from the dish when they reached 90-100% confluence using 1x Trypsin/EDTA (Gibco; 15400).

Cell viability and survival

**Figure 1:**
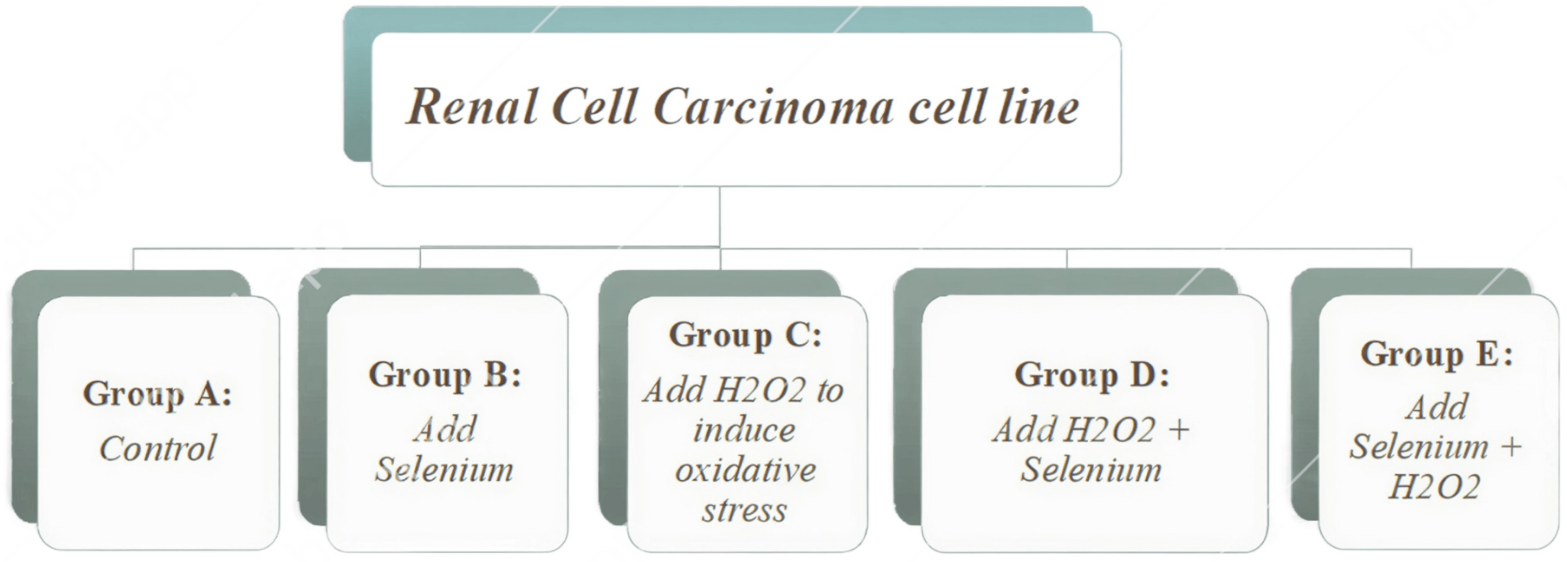
Renal cell carcinoma cell line division

As depicted in Figure 1, renal cell carcinoma cell lines were divided into two groups: control and experimental groups. Group A served as the control with no additions. Experimental groups were divided into groups from B to E. Group B was treated with selenium (Sigma-Aldrich; 229865). Oxidative stress was induced in group C after 24 hours using hydrogen peroxide solution (Sigma; H1009). Oxidative stress was also induced in Group D using hydrogen peroxide, followed by treatment with selenium. Group E was treated with selenium treatment followed by the induction of oxidative stress by H₂O₂. All the cells were counted, and 40000 cells were plated on a flat-bottom 96-well plate.

Thiazolyl Blue Tetrazolium Bromide (Sigma; M2128) was added after removing the medium, and the readings were taken at 540 nm. Statistical analysis was performed using the chi-square test.

Cellular reactive oxygen species (ROS) levels assay

Cellular reactive oxygen species detection assay kit (Abcam; ab186029) was used to quantify intracellular ROS levels. RCC cells were harvested and counted, and 40,000 cells per well were plated in flat-bottom 96-well plates and allowed to adhere overnight in Dulbecco’s Modified Eagle’s Medium (DMEM; Gibco, 61965). A density of 40,000 cells/well was selected, as it yields optimal 60-70% confluency for reliable fluorescence detection and reproducible ROS and viability measurements in a 96-well format.

Selenium Preparation and Treatment

Selenium was used in the form of sodium selenite (Na₂SeO₃). A stock solution was prepared in sterile distilled water and filtered through a 0.22 µm membrane. Cells were treated with 5 µM sodium selenite for 24 hours for the selenium-only group (Group B). This same concentration and exposure duration were applied to groups receiving selenium before or after oxidative stress induction (Groups D and E).

Hydrogen Peroxide (H₂O₂) Preparation and Oxidative Stress Induction

Oxidative stress was induced using hydrogen peroxide (H₂O₂). A fresh working solution was prepared immediately before use. Cells were exposed to 200 µM H₂O₂ for 2 hours for the oxidative-stress group (Group C). The same concentration and duration were used for groups treated with selenium followed by H₂O₂ (Group E) or H₂O₂ followed by selenium (Group D).

Treatment Sequence

Group A (Control): No selenium or H₂O₂ treatment. Group B (Selenium only): Selenium for 24 hours. Group C (H₂O₂ only): H₂O₂ for 2 hours. Group D (H₂O₂ → Selenium): H₂O₂ for 2 hours, followed by 24-hour selenium treatment. Group E (Selenium → H₂O₂): Selenium for 24 hours, followed by 2-hour H₂O₂ exposure.

After treatments, assay buffers were added according to the manufacturer’s instructions. The ROS-sensitive working solution was added to each well and incubated for 30-60 minutes at 37°C. Fluorescence readings were taken at Ex/Em = 650/675 nm.

Although MTT absorbance and ROS fluorescence were originally measured as continuous values, these data were categorised into outcome groups (e.g., increased vs. decreased viability; low vs. high ROS) based on predefined biological thresholds. Because the final dataset consisted of categorical variables, chi-square testing was used to evaluate differences in proportions between the study groups. This approach is appropriate for categorical data and does not require assumptions of normality or homogeneity of variance. Statistical significance was set at p < 0.05.

## Results

After each group was treated with its assigned protocol, the ROS production in each group was measured. In addition, the cell viability was measured using the MTT kit assay. 

All results were entered and evaluated using statistical analysis performed using IBM Corp. Released 2016. IBM SPSS Statistics for Windows, Version 22. Armonk, NY: IBM Corp. to calculate the p-values for the chi-square test.

**Figure 2:**
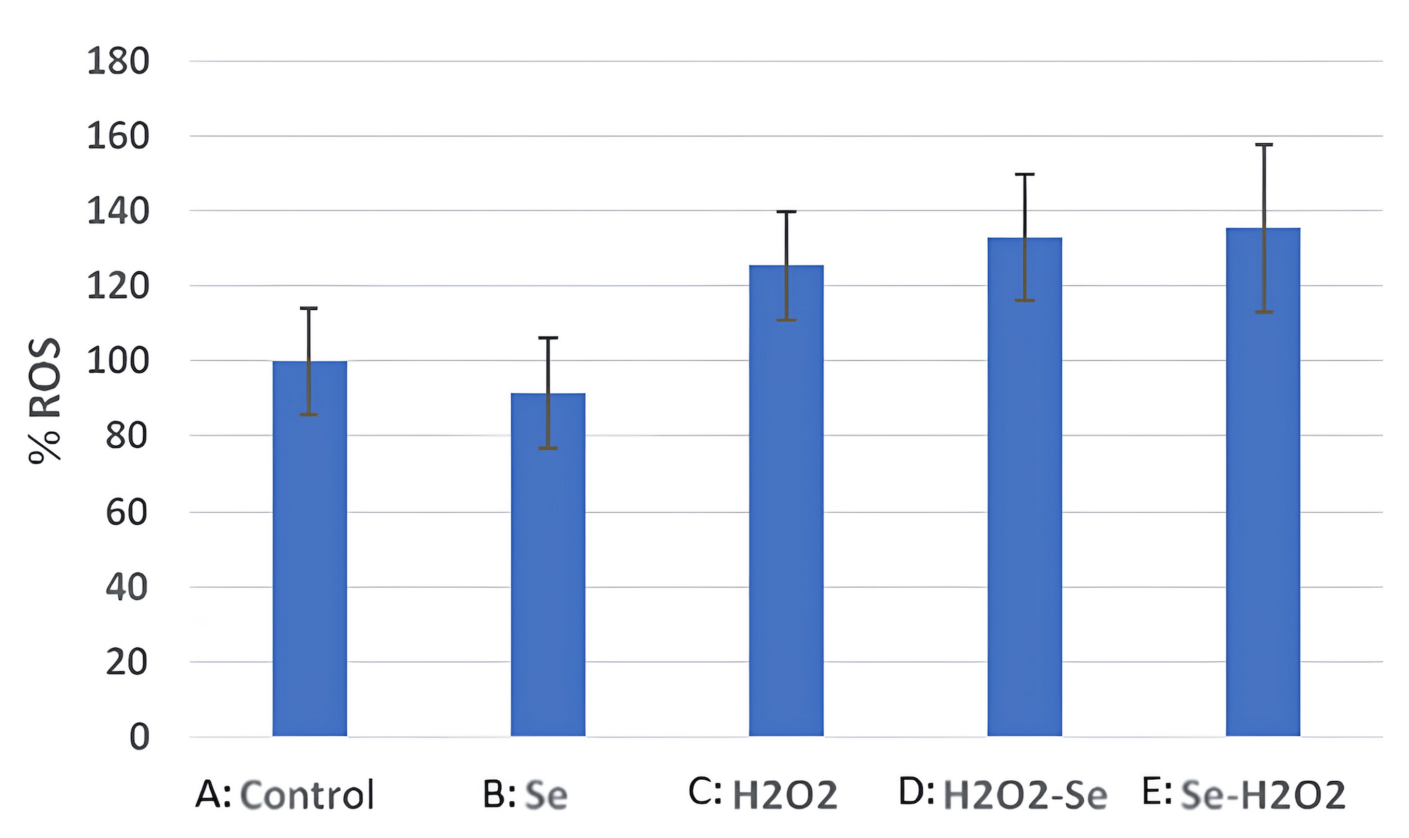
This graph demonstrates the percentage of reactive oxygen species (ROS) produced among the different groups

As depicted in Figure 2, the generation of the ROS was variable among the different groups, with the highest levels being observed in group E (Se-H₂O₂, 135.3% ± 22.1), followed by group D (H₂O₂-Se, 132.9% ± 16.6), and group C (H₂O₂, 125.4% ± 14.6). The two groups that showed the lowest levels included group A (Control, 100% ± 14) and group B (Se, 91.7% ± 14.6).

Group D (H₂O₂ → Se)

Cells were first exposed to H₂O₂ for 2 hours to induce oxidative stress. Following H₂O₂ removal, the wells were washed once with PBS, and fresh medium containing selenium (sodium selenite) was added for 24 hours. No overlap between treatments occurred; the selenium exposure began only after completion of the H₂O₂ exposure period.

Group E (Se → H₂O₂)

Cells were first treated with selenium (sodium selenite) for 24 hours. After incubation, the selenium-containing medium was removed, the wells were washed once with PBS, and fresh medium containing H₂O₂ was applied for 2 hours. As with Group D, treatments were sequential and non-overlapping.

**Figure 3:**
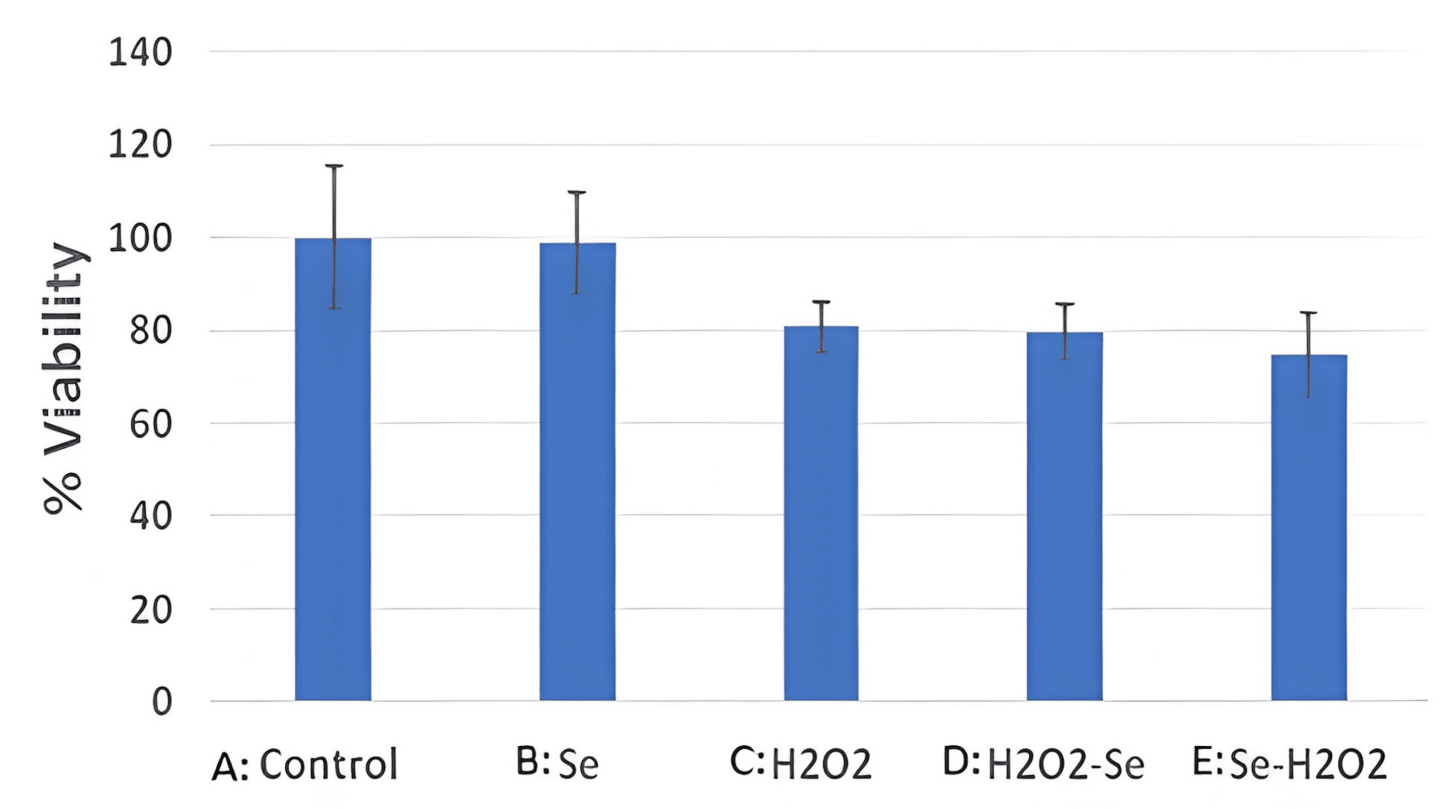
This graph demonstrates the percentage of viable renal cell carcinoma cells between different groups

Figure 3 shows the difference in the viability of cells among different groups. The lowest levels of viability were observed in group E (Se-H₂O₂, 74.8% ± 8.8), followed by group D (H₂O₂-Se, 79.7% ± 5.9) and group C (H₂O₂, 80.8% ± 5.4). On the other hand, Group B (Se, 98.7% ± 10.8) showed the highest levels of cell viability, followed by Group A (Control, 100% ± 14).

The ROS generation, as illustrated in Table 1, in Groups C, D, and E was significantly high (p-value = 0.008; p-value = 0.007; p-value = 0.003, respectively) when compared to the control group, A. On the other hand, group B had lower ROS production than group A. However, the difference was not statistically significant. Whereas when compared to group B, groups C, D, and E showed significantly higher levels (p-value = 0.002; p-value = 0.001; p-value = 0.0009, respectively).

**Table 1:** Percentage of Reactive Oxygen Species (ROS) Production

Group	Result		p-value
A-C	A: 100% ± 14	C: 125.4% ± 14.6	0.008
A-D	A: 100% ± 14	D: 132.9% ± 16.6	0.007
A-E	A: 100% ± 14	E: 135.3% ± 22.1	0.003
B-C	B: 91.7% ± 14.6	C: 125.4% ± 14.6	0.002
B-D	B: 91.7% ± 14.6	D: 132.9% ± 16.6	0.001
B-E	B: 91.7% ± 14.6	E: 135.3% ± 22.1	0.0009

As for cell viability, as illustrated in Table 2, groups C, D, and E showed significantly lower levels (p-value = 0.01; p-value = 0.003; p-value = 0.006, respectively) when compared to the control group A. ROS production of group B was lower than group A. However, the difference was not statistically significant. However, groups C, D, and E showed significantly lower levels of ROS when compared to group B (p-value = 0.005; p-value = 0.0009; p-value = 0.002, respectively).

**Table 2:** MTT levels reflecting amount of cell viability

Group	Result		p-value
A-C	A: 100% ± 14	C: 80.8% ± 5.4	0.01
A-D	A: 100% ± 14	D: 79.7%± 5.9	0.003
A-E	A: 100% ± 14	E: 74.8% ± 8.8	0.006
B-C	B: 98.7% ± 10.8	C: 80.8% ± 5.4	0.005
B-D	B: 98.7% ± 10.8	D: 79.7%± 5.9	0.0009
B-E	B: 98.7% ± 10.8	E: 74.8% ± 8.8	0.002

## Discussion

Our findings demonstrate clear differences in cell viability among the RCC groups treated with selenium, hydrogen peroxide, or their combination. A consistent pattern emerged: higher ROS levels were associated with lower cell viability, whereas lower ROS levels were associated with higher viability. This supports the widely accepted concept that excessive ROS can overwhelm cellular metabolic capacity, leading to oxidative damage and reduced survival. Glasauer et al. (2014) similarly reported that high ROS burdens can be cytotoxic to cancer cells and induce cell death [4].

In the control group (A), the highest cell viability and baseline ROS levels were observed, reflecting the untreated physiological state of the RCC cells. Selenium alone (group B) produced the lowest ROS levels and the second-highest viability, supporting the classical antioxidant role of selenium, which, through its incorporation into selenoproteins, can neutralise ROS and protect cells from oxidative injury. This aligns with prior literature describing selenium’s capacity to reduce ROS and improve cellular redox balance [4].

In contrast, all groups exposed to hydrogen peroxide (C, D, and E) showed lower viability than the control or selenium-only groups, consistent with the expected cytotoxicity from ROS overload. Group C (H₂O₂ alone) demonstrated markedly increased ROS and a corresponding drop in viability, confirming successful oxidative stress induction.

The combined-treatment groups (D and E) were used to explore whether selenium could either prevent (Se → H₂O₂) or repair damage (H₂O₂ → Se) induced by ROS. Contrary to expectations, both groups showed higher ROS levels and lower viability than selenium alone, and even more notably, worse viability than H₂O₂ alone. This paradoxical effect suggests two possible explanations.

First, selenium may lose its antioxidant function under conditions of excessive oxidative stress. When ROS production exceeds a critical threshold, selenium-dependent antioxidant enzymes may become overwhelmed or inactivated, limiting their protective effect. Second, selenium is well known to exhibit dose- and context-dependent pro-oxidant behaviour, particularly in the presence of high ROS. Under such conditions, selenium compounds can shift from acting as antioxidants to functioning as oxidants, thereby amplifying oxidative damage rather than reducing it. This phenomenon is consistent with the dual, “double-edged sword” nature of selenium described in cancer biology.

The worsening viability in groups D and E compared with group C therefore suggests that selenium, when combined with high ROS levels, may have switched from an antioxidant to a pro-oxidant, exacerbating oxidative stress instead of mitigating it.

Overall, our results indicate a strong association between ROS generation and RCC cell viability. Groups with elevated ROS (C, D, E) exhibited reduced viability, whereas the group with the lowest ROS (B) showed the greatest viability improvement. These findings support the concept that selenium’s effects on RCC cells are highly context-dependent and may become harmful in environments dominated by oxidative stress.

Study limitations

Several limitations of this study should be acknowledged to contextualise the findings. First, although we investigated the effect of selenium under different oxidative states, only one RCC cell line was used, which limits the generalisability of the results across RCC subtypes or in vivo tumour environments. Second, selenium’s biological effects are known to be dose-dependent and context-dependent, with evidence supporting both its antioxidant and pro-oxidant roles. While our findings suggest a possible shift toward pro-oxidant activity under high ROS conditions, this interpretation must be made with caution and has now been supported with relevant literature. Third, the study did not include mechanistic assays (e.g., caspase activation, mitochondrial membrane potential, selenoprotein expression), and therefore no definitive conclusions can be drawn about the specific pathways linking selenium, ROS, and cell death. Elevated ROS levels observed in groups D and E indicate an association with reduced viability but do not establish causation in the absence of mechanistic validation.

Fourth, the statistical analysis is limited by the need to categorise continuous MTT and ROS measurements into discrete groups for chi-square testing. Although this approach is now fully explained and justified, it inherently simplifies the data and may reduce sensitivity compared to continuous-variable statistical models. Finally, while all experimental parameters (selenium concentration, H₂O₂ concentration, exposure times, replicates, and treatment sequences) have now been clearly reported, their absence in the initial submission represents an important methodological limitation.

Despite these limitations, the study provides meaningful preliminary insight into selenium’s dual redox-dependent behaviour in RCC cells and highlights the importance of oxidative context in determining whether selenium supports or impairs cancer cell survival. Future work incorporating multiple cell lines, dose-response curves, and mechanistic assays is needed to clarify selenium’s precise mode of action.

## Conclusions

In conclusion, the effects of selenium as an antioxidant in promoting cell viability in renal cell carcinoma have been controversial. In some aspects, it reduced ROS production that increased cancer cell viability. While in another setting, selenium increased ROS levels that correlated with more cell death. Therefore, further research needs to be conducted to prove the true effects of selenium on cancer cells.
